# Synthesis of isopropyl-substituted anthraquinones via Friedel–Crafts acylations: migration of isopropyl groups

**DOI:** 10.1098/rsos.170451

**Published:** 2017-08-23

**Authors:** Abdel B. Chakiri, Philip Hodge

**Affiliations:** Department of Chemistry, University of Manchester, Oxford Road, Manchester M13 9PL, UK

**Keywords:** quinone, synthesis, rearrangements

## Abstract

Friedel–Crafts reactions of isopropyl-substituted benzenes with phthalic anhydride in the presence of aluminium trichloride, followed by cyclization of the products with strong sulfuric acid give, as expected, anthraquinones. The syntheses, however, often afford more than one anthraquinone. In some cases the isopropyl groups migrate cleanly to other ring positions; in other cases they are lost.

## 1. Introduction

Hydrogen peroxide is a very important green oxidant because in many applications the only by-product is water [1]. Most hydrogen peroxide is manufactured by the Riedel–Pfleiderer process [2]. This involves hydrogenating an alkylanthraquinone to give the corresponding alkyldihydroxyanthracene, then the reaction of this with oxygen or air to give hydrogen peroxide. The latter reaction also regenerates the alkylanthraquinone which can then be recycled. The alkyl group is needed to improve the solubilities of the alkylanthraquinone and the corresponding alkyldihydroxyanthracene in the reaction solvent(s). High solubilities are crucial to the efficiency of the overall process [2].

In this connection we have investigated the synthesis of several isopropyl-substituted anthraquinones. The classical two-step approach was used (e.g. [3,4]). Thus, (i) an isopropyl-substituted benzene was subjected to a Friedel–Crafts acylation using phthalic anhydride in the presence of aluminium trichloride, then (ii) the benzoylbenzoic acid(s) formed was/were cyclized with strong sulfuric acid. We now report that syntheses starting with isopropyl-substituted benzenes generally produce more than one anthraquinone. In some cases the isopropyl groups migrate efficiently and cleanly to other ring positions; in other cases they are lost.

There appears to be no literature examples of isopropyl groups migrating during Friedel–Crafts acylations using phthalic anhydride, but acetylations of 1,4-diisopropylbenzene [5] and 3- and 4-isopropyltoluene [6] that involve isopropyl migrations have been reported. Occasionally alkyl migrations have been reported in Friedel–Crafts alkylations [7].

## 2. Results

The present syntheses were carried out using standardized procedures. Thus, Friedel–Crafts reactions of the isopropyl-substituted benzenes with phthalic anhydride (equimolar amounts) in the presence of aluminium trichloride (2.1 molar equivalents) were carried out in dichloromethane at 42°C for 30 min. The aromatic hydrocarbon was added last. The products were cyclized by treatment with 4% oleum at 95°C for 2 h. Crude yields of anthraquinones were 48–63% overall. Pure anthraquinones were isolated via column chromatography and carefully characterized. ^1^H NMR spectroscopy was particularly helpful in this context as protons in or attached to substituents in α-positions of anthraquinones resonate downfield from similar protons in or attached to substituents in β-positions: see the electronic supplementary material.

### 2.1. Anthraquinone synthesis starting with 1,3-diisopropylbenzene

Reaction of 1,3-diisopropylbenzene with phthalic anhydride and aluminium trichloride gave a mixture of benzoylbenzoic acids (*ca* 94% yield). By ^1^H NMR spectroscopy it consisted of acids **1** and **2** in a ratio of 10 : 1. The latter was identified by comparison with the spectrum of an authentic sample [4] (see the electronic supplementary material). Cyclization of the mixture of acids gave a mixture of anthraquinones (59% yield). ^1^H NMR spectroscopy indicated the latter consisted of 1,3-diisopropylanthraquinone (**3**) and 2-isopropylanthraquinone (**4**) in a ratio of 10 : 1. These were isolated via column chromatography and characterized. Anthraquinone **3** was obtained from the cyclization in 54% yield and anthraquinone **4** in 5% yield, i.e. a mole ratio of 11 : 1. Clearly, the cyclization step did not significantly alter the proportions of the diisopropyl and isopropyl products. In summary, this quinone synthesis proceeds essentially as expected.


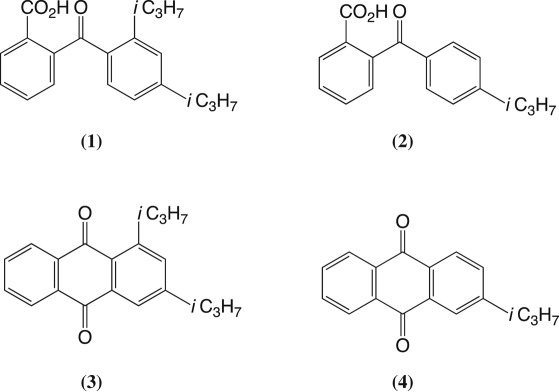


### 2.2. Anthraquinone synthesis starting with 1,4-diisopropylbenzene

Reaction of 1,4-diisopropylbenzene with phthalic anhydride and aluminium trichloride surprisingly gave, by ^1^H NMR spectroscopy of the crude product, a mixture of the same two benzoylbenzoic acids (*ca* 94% yield) as were obtained in the previous synthesis, i.e. acids **1** and **2**, this time in the ratio of 3.1 : 1. The isolated products were again anthraquinones **3** (36% yield from the cyclization) and **4** (12%): i.e. mole ratio of 3.0 : 1. Thus, as before, the cyclization did not significantly affect the proportions of isopropyl and diisopropyl products.

### 2.3. Anthraquinone synthesis starting with 3-isopropyltoluene

3-Isopropyltoluene (*meta*-cymene) was treated with phthalic anhydride and aluminium trichloride and the product immediately cyclized to give crude anthraquinone(s) (63% overall). Column chromatography gave 1-methyl-3-isopropylanthraquinone (**5**) in 56% isolated yield overall and 2-methylanthraquinone (**6**) in 4% yield. No 2-isopropylanthraquinone (**4**) was found.

### 2.4. Anthraquinone synthesis starting with 4-isopropyltoluene

4-Isopropyltoluene (*para*-cymene) was reacted with phthalic anhydride and aluminium trichloride and the product immediately cyclized using the usual procedures. This gave crude anthraquinones in 62% overall yield. This time column chromatography afforded anthraquinones **5**, **6** and **7** in overall yields of 35%, 4% and 23% respectively. No 2-isopropylanthraquinone (**4**) was found.


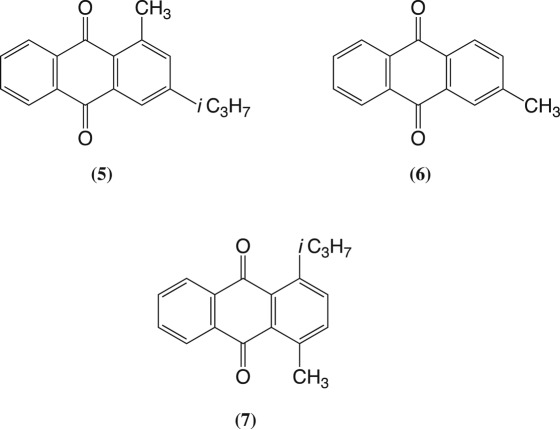


Anthraquinone **7** has been prepared before in the authors' laboratory via the Diels–Alder reaction of 1,4-naphthoquinone with α-terpinene [8], and the present product was identified by direct comparison with the previous product.

The Friedel–Crafts reaction of 4-isopropyltoluene with phthalic anhydride followed by cyclization has been reported twice before to give just anthraquinone **7** [9,10]. However, in neither case was strong evidence produced to support this structure. The products described in the literature had m.p. of 113.8°C [9] and 114°C [10]. These are significantly higher than the m.p. of 79–81°C and 90–92°C we find for quinones **5** and **7**, respectively. Thus, the structure(s) of the products described previously are unclear.

## 3. Discussion of results

It is clear from the above results that migration of isopropyl groups occurs during the acylation of 1,4-diisopropylbenzene and 4-isopropyltoluene, but not in the acylation of the corresponding 1,3-isomers. During the acylation reactions of both diisopropyl isomers and both isopropylmethyl isomers some loss of isopropyl groups occurs leading ultimately to the formation of 2-isopropyl- (**4**) or 2-methyl-anthraquinone (**6**) respectively. Dealkylation reactions are well known to occur during electrophilic substitutions of benzenoid compounds [11].

**Scheme 1.**
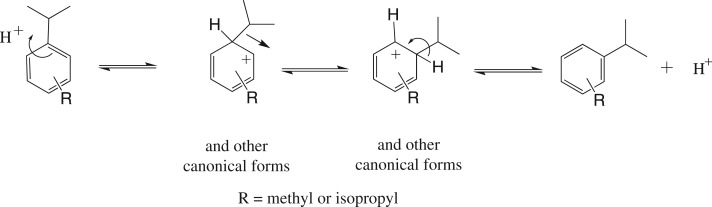
Acid-catalysed *intramolecular* equilibration of alkylbenzenes. The migration may also occur to some extent *intermolecularly* by dealkylation (scheme 2) followed by realkylation [12,13].

**Scheme 2.**
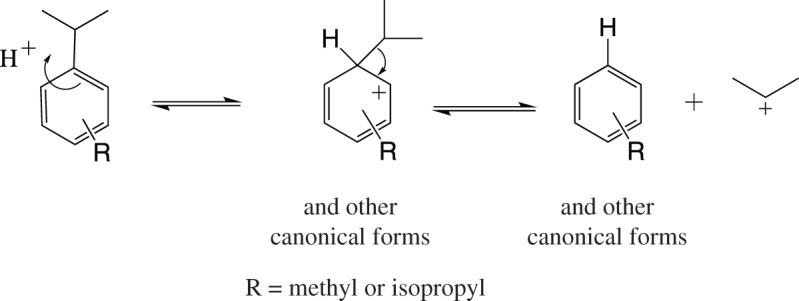
Acid-catalysed dealkylation of alkylbenzenes [11].

The migration and dealkylation reactions almost certainly occur as outlined in schemes 1 and 2 respectively. Both involve electrophilic attack by protons on the aromatic rings [12,13]. The *catalytic* amount of acid required is generated as a by-product of the initial acylation, and, possibly also, by traces of moisture reacting with the aluminium trichloride. The hydrocarbon starting materials are clearly very much more prone to such electrophilic reactions than the benzoylbenzoic acid(s) produced by the acylation. In the subsequent acid-catalysed cyclization the final anthraquinone products will be even less reactive to electrophiles. Thus, the migration almost certainly occurs only at the very first stage of the quinone synthesis. It is clear the proportions of the acylation and dealkylation products from the two diisopropylbenzenes do not change significantly during treatment with sulfuric acid to achieve cyclization.

It is clear that when the aromatic hydrocarbon is added to the acylation mixture there will be a complex competition between several reactions: (i) acylation(s), (ii) intramolecular migration, (iii) intermolecular migration, and (iv) dealkylation. When phthalic anhydride is used, as in the present work, the active acylating species is significantly more sterically demanding than the corresponding species from acetic anhydride or acetyl chloride and hence it is expected to react less rapidly. This gives the migration and dealkylation reactions a better chance of competing significantly.

Reactions of the type shown in scheme 1 have been studied before, and it has been shown that both 1,3- and 1,4-diisopropylbenzene [12] and both 1,3- and 1,4-isopropyltoluene [13] equilibrate readily. Since in addition it is expected that the 1,3-isomers will be more reactive to acylation (the aromatic position next to an isopropyl or methyl substituent is activated by one *ortho*- and one *para*-alkyl group whereas with the 1,4-isomer it is activated by one *ortho*- and one *meta*-alkyl group), it is clear that the 1,4-isomers are the more likely to become involved in migrations and dealkylations. It is not suggested that in the present reactions the 1,4-isomers fully equilibrate before acylation begins.

With 1,3-diisopropylbenzene and 1,3-isopropyltoluene acylation proceeds smoothly at the positions *ortho* to an isopropyl or the methyl substituent resulting in quinones **3** and **5** respectively. There is no evidence that the reactions shown in scheme 1 play a significant part.

With 1,4-diisopropylbenzene and 4-isopropyltoluene the reactions summarized in scheme 1 compete with acylation and rearranged product is acylated as before to give quinones **3** and **5** respectively. However, with 4-isopropyltoluene a substantial amount of quinone **7** is also formed. This may be formed via direct acylation of the hydrocarbon *ortho* to the methyl substituent and/or via the reactions as shown in scheme 1.

Finally, when dealkylation occurs, as expected [11], it is always an isopropyl group that is lost, not a methyl group.

## 4. Conclusion

Synthesis of anthraquinones by Friedel–Crafts reactions of isopropyl-substituted benzenes with phthalic anhydride, followed by cyclization of the products using strong sulfuric acid can give mixtures of anthraquinones. With 1,3-diisopropylbenzene the main product is 1,3-diisopropylanthraquinone (**3**) accompanied by 2-isopropylanthraquinone (**4**). With 1,4-diisopropylbenzene efficient migration of an isopropyl substituent occurs and the main products are again 1,3-diisopropylanthraquinone (**3**) and 2-isopropylanthraquinone (**4**). With 3-isopropyltoluene the main isolated product is 1-methyl-3-isopropylanthraquinone (**5**) together with minor amount of 2-methylanthraquinone (**6**). With 4-isopropyltoluene anthraquinones **5**, **6** and **7** were isolated. In this latter case the formation of **5** involves rearrangement.

## 5. Experimental

### 5.1 General details

Chemicals were purchased from Sigma-Aldrich®, Fluka or Alfa Aesar®.

Products were dried in a vacuum oven at 40°C and 5 mm pressure. Melting points were determined using a Barloworld Scientific SMP10 melting point analyser. Fourier transform infrared (FT-IR) spectra were recorded on a Thermo Scientific Nicolet iS5 FT-IR spectrometer. Elemental analyses were performed in house by Martin Jennings using a Carlo Erba Instruments EA1108 elemental analyser.

^1^H NMR spectra were recorded for solutions in deuterated chloroform with either a B400 Bruker Avance III 400 MHz or a B500 Bruker Avance II+ 500 MHz spectrometer. Abbreviations: singlet (s), doublet (d), septet (sept), doublet of doublets (dd), triplet of doublets (td), multiplet (m). Coupling constants (*J*) are reported in hertz (Hz).

### 5.2. General procedure for Friedel–Crafts reactions: reaction of 1,3-diisopropylbenzene with phthalic anhydride

Phthalic anhydride (68.0 g, 0.44 mol) was dissolved in dry dichloromethane (800 ml) and aluminium trichloride (128.5 g, 0.96 mol) was added. The initial white suspension soon became a yellow solution. This was stirred for 30 min at room temperature, then cooled to 0°C and 1,3-diisopropylbenzene (74.0 g, 0.46 mol) added dropwise. After the addition was complete the mixture was allowed to warm up to room temperature (1 h) with stirring and was then heated under reflux for 30 min. The final reaction mixture was cooled and poured into a mixture of crushed ice and dilute hydrochloric acid. The aqueous layer was extracted with dichloromethane, the organic layers combined, dried over anhydrous magnesium sulfate and concentrated under reduced pressure to give crude benzoylbenzoic acids (127.8 g; *ca* 94%), m.p. 104–107°C; FT-IR ν_max_ (ATR): 2962, 1693, 1666 and 1602 cm^−1^. The crude product was shown by ^1^H NMR spectroscopy to be mainly acid **1** (90%): ^1^H NMR (500 MHz, CDCl_3_) *δ*_ppm_: 7.99 (d, ^3^*J*_HH_ = 7.1 Hz, 1H), 7.60 (td, ^3^*J*_HH_ = 7.5 Hz, ^4^*J*_HH_ = 1.2 Hz, 1H), 7.55 (td, ^3^*J*_HH_ = 7.6 Hz, ^4^*J*_HH_ = 1.0 Hz, 1H), 7.40 (d, ^3^*J*_HH_ = 7.6 Hz, 1H), 7.31 (d, ^4^*J*_HH_ = 1.3 Hz, 1H), 7.09 (d, ^3^*J*_HH_ = 8.1 Hz, 1H), 6.94 (dd, ^3^*J*_HH_ = 8.1 Hz, ^4^*J*_HH_ = 1.5 Hz, 1H), 3.82 (sept, ^3^*J*_HH_ = 6.8 Hz, 1H), 2.91 (sept, ^3^*J*_HH_ = 6.9 Hz, 1H), 1.26 (d, ^3^*J*_HH_ = 6.8 Hz, 6H), 1.24 (d, ^3^*J*_HH_ = 6.8 Hz, 6H). The spectrum also showed signals due to the presence of acid **2** (9%) at *δ*_ppm_: 8.08 (dd, ^3^*J*_HH_ = 7.9 Hz, ^4^*J*_HH_ = 0.8 Hz, 1H), 7.66 (d, ^3^*J*_HH_ = 8.4 Hz, 2H), 7.65 (td, ^3^*J*_HH_ = 6.5 Hz, ^4^*J*_HH_ = 1.2 Hz, 1H), 7.56 (td, ^3^*J*_HH_ = 7.7 Hz, ^4^*J*_HH_ = 1.1 Hz, 1H), 7.36 (dd, ^3^*J*_HH_ = 7.5 Hz, ^4^*J*_HH_ = 0.8 Hz, 1H), 7.28 (s, 1H), 2.95 (sept, ^3^*J*_HH_ = 6.9 Hz, 1H), 1.26 (d, ^3^*J*_HH_ = 6.9 Hz, 6H).

Other phthaloylations were carried out similarly albeit on different scales.

### 5.3. General procedure for cyclization to anthraquinones: cyclization of the crude benzoylbenzoic acids prepared from 1,3-diisopropylbenzene

A portion of the above crude acid (70.0 g, 0.23 mol) was dissolved in 4% oleum (1.2 molar equivalent sulfur trioxide). The solution was stirred at 95°C for 2 h and was then allowed to cool down to room temperature. Water was added (10 portions of 5 ml) carefully and the mixture extracted with dichloromethane (25 ml portions) until the extracts were no longer yellow. The combined organic layers were washed with water, dried over anhydrous magnesium sulfate and concentrated under reduced pressure to give a mixture of anthraquinones (38.9 g, *ca* 59%). By ^1^H NMR spectroscopy (in comparison with authentic samples of quinones) it contained quinones **3** and **4** in a ratio of 10 : 1. Column chromatography (silica gel, eluent = dichloromethane : hexane (1 : 9)) gave samples of two anthraquinones. First, anthraquinone **3** (54% yield from the mixture of benzoylbenzoic acids) as yellow crystals, m.p. 69–70°C; FT-IR ν_max_ (ATR): 2961, 2869 and 1664 cm^−1^; ^1^H NMR (500 MHz, CDCl_3_) *δ*_ppm_: 8.26–8.21 (m, 2H), 8.12 (d, ^4^*J*_HH_ = 1.8 Hz, 1H), 7.80–7.70 (m, 2H), 7.65 (d, ^4^*J*_HH_ = 1.8 Hz, 1H), 4.47 (sept, ^3^*J*_HH_ = 6.8 Hz, 1H), 3.05 (sept, ^3^*J*_HH_ = 6.9 Hz, 1H), 1.35 (d, ^3^*J*_HH_ = 6.8 Hz, 6H), 1.33 (d, ^3^*J*_HH_ = 6.9 Hz, 6H). This NMR spectrum is shown in the electronic supplementary material, figure S1. Elemental analysis calculated for C_20_H_20_O_2_: C, 82.16%; H, 6.89%. Found: C, 82.14%; H, 6.90%. Second, 2-isopropylanthraquinone (**4**) (5% yield); ^1^H NMR (500 MHz, CDCl_3_) *δ*_ppm_: 8.35–8.27 (m, 2H), 8.24 (d, ^3^*J*_HH_ = 8.0 Hz, 1H), 8.17 (d, ^4^*J*_HH_ = 1.7 Hz, 1H), 7.82–7.76 (m, 2H), 7.66 (dd, ^3^*J*_HH_ = 8.0 Hz, ^4^*J*_HH_ = 1.7 Hz, 2H), 3.10 (sept, ^3^*J*_HH_ = 6.9 Hz, 1H), 1.34 (d, ^3^*J*_HH_ = 7.0 Hz, 6H) identified by m.p., mixed m.p., FT-IR and ^1^H NMR spectra in comparison with an authentic sample (see electronic supplementary material, figure S2). Thus, the ratio of isolated non-dealkylated product to isolated alkylated product was 10.8 : 1.

Other cyclizations were carried out similarly, albeit on different scales.

### 5.4. Friedel–Crafts acylation of 1,4-diisopropylbenzene

Phthalic anhydride (85.9 g), aluminium trichloride (170 g) and 1,3-diisopropylbenzene (100 g) were reacted together using the general procedure. The crude acidic product (170 g, *ca* 94%) had m.p. 44–60°C; FT-IR ν_max_ (ATR): 2959, 1678, 1668 and 1604 cm^−1^. The ^1^H NMR spectrum (500 MHz, CDCl_3_) indicated that, as above, the product contained acids **1** and **2**, but now in a ratio of 3.1 : 1.

### 5.5. Cyclization of benzoylbenzoic acids prepared from 1,4-diisopropylbenzene

Crude acid (65 g) prepared above was cyclized using the general procedure. This gave a mixture of anthraquinones (33.6 g, *ca* 51%). Column chromatography gave two anthraquinones. First, anthraquinone **3** (36% yield from crude benzoylbenzoic acids) as yellow crystals, m.p. 69°C; FT-IR and ^1^H NMR spectra the same as reported above. Second, anthraquinone **4** (12% yield) as yellow crystals, m.p. 44°C; FT-IR and ^1^H NMR spectra same as reported above. Thus, the ratio of non-dealkylated product to alkylated product was 3.0 : 1.

### 5.6. Synthesis of anthraquinones from 3-isopropyltoluene

Phthalic anhydride (12.0 g), aluminium trichloride (24.0 g), and 3-isopropyltoluene (11.1 g) were reacted together using the above general procedure. The crude product (20.2 g) was cyclized without further purification. This gave a mixture of anthraquinones (*ca* 63% yield). Column chromatography gave anthraquinone **5** (56% overall yield) with m.p. 79–81°C; FT-IR ν_max_ (ATR): 2962, 1693, 1666 and 1602 cm^−1^; ^1^H NMR (400 MHz, CDCl_3_) *δ*_ppm_: 8.20 (dd, ^3^*J*_HH_ = 7.0 Hz, ^4^*J*_HH_ = 1.1 Hz, 2H), 8.07 (s, 1H), 7.75–7.67 (m, 2H), 7.38 (s, 1H), 3.00 (sept, ^3^*J*_HH_ = 6.9 Hz, 1H), 2.80 (s, 3H), 1.30 (d, ^3^*J*_HH_ = 7.0 Hz, 6H): see electronic supplementary material figure S3. Elemental analysis: expected for C_18_H_16_O_2_; C = 81.8, H = 6.1%: found C = 82.3 and H = 5.9%. A second anthraquinone was identified as 2-methylanthraquinone (**6**; 4%), m.p. 174–175°C (lit. 176°C); FT-IR ν_max_ (ATR): 3063, 3029, 3009, 1673, 1592 cm^−1^; ^1^H NMR (400 MHz, CDCl_3_) *δ*_ppm_: 8.35–8.26 (m, 2H), 8.20 (d, ^3^*J*_HH_ = 7.9 Hz, 1H), 8.10 (s, 1H), 7.83–7.75 (m, 2H), 7.59 (d, ^3^*J*_HH_ = 7.9 Hz, 1H), 2.54 (s, 3H): see electronic supplementary material, figure S4. No 2-isopropylanthraquinone (**4**) was found.

### 5.7. Synthesis of anthraquinones from 4-isopropyltoluene

Phthalic anhydride (12.0 g), aluminium trichloride (24.0 g) and 4-isopropyltoluene (11.1 g) were reacted together using the standard procedure. This gave crude acids (21.4 g, *ca* 95% yield), a portion (10.0 g) of which was cyclized using the standard procedure. Column chromatography of the crude product (65%) afforded three anthraquinones. First, anthraquinone **5** (35% yield overall), data as above. Second, anthraquinone **7** (23%) with m.p. 90–92°C (lit. 114°C); FT-IR ν_max_ (ATR): 2952, 2924, 2865, 1659, 1592 cm^−1^; ^1^H NMR (400 MHz, CDCl_3_) *δ*_ppm_: 8.39–8.30 (m, 2H), 7.97–7.90 (m, 2H), 7.88 (d, ^3^*J*_HH_ = 8.2 Hz, 1H), 7.71 (d, ^3^*J*_HH_ = 8.2 Hz, 1H), 4.46 (sept, ^3^*J*_HH_ = 6.8 Hz, 1H), 3.01 (s, 3H), 1.54 (d, ^3^*J*_HH_ = 6.8 Hz, 6H): see electronic supplementary material figure S5. Elemental analysis: expected for C_18_H_16_O_2_; C = 81.8, H = 6.1%: found C = 81.1 and H = 6.3%. Third, anthraquinone **6** (4%), m.p. 174–175°C, spectroscopic data as above. No 2-isopropylanthraquinone (**4**) was found.

## Supplementary Material

As paper

## Data accessibility

Further details are available in the electronic supplementary material. This includes further experimental details and various NMR spectra.
