# Supplementary material for: Synthesis of isopropyl-substituted anthraquinones via Friedel–Crafts acylations: migration of isopropyl groups
Source: R Soc Open Sci. 2017 Aug 23;4(8):170451. doi: 10.1098/rsos.170451 (PMC5579107; doi:10.1098/rsos.170451)

ESM for

Synthesis of Isopropyl-substituted Anthraquinones via Friedel-Crafts Acylations: Migration of Isopropyl Groups.

Abdel B. Chakiri and Philip Hodge*

Department of Chemistry, University of Manchester, Oxford Road,

Manchester M13 9PL UK

Compound formulae and reference numbers are the same as, or follow on from, those given in the main paper.

This ESM considers the ^{1}H NMR spectra of anthraquinone derivatives. The spectra of anthraquinones **3** – **7** are included. Experimental details for the preparation of authentic samples of 2-isopropylanthraquinone (**4**) and the intermediate 2-benzoylbenzoic acid (**2**) are given.

1) ^{1}H NMR Spectra of anthraquinones

All the following spectra were measured for solutions in $CDCl_3$ on the instruments reported in the main paper.

In the ^{1}H NMR spectra of anthraquinones the α- and β-hydrogens resonate at significantly different positions, as do the α- and β-methyl groups, and the methine protons in α- and β-isopropyl groups. Thus, in anthraquinone itself (available commercially) the α-protons resonate at δ 8.32 ppm whereas the β-protons resonate at δ 7.81 ppm. In 2-methylanthraquinone (**6**) (available commercially) the methyl group resonates at δ 2.54 ppm: see Figure 4. In 2-isopropylanthraquinone (**4**) (prepared as below) the methyl groups resonate as doublet at δ 1.34 ppm, whereas the methine proton resonates at δ 3.10 ppm: see Figure 2. In 4-methyl-1-isopropylanthraquinone (**7**) (available from previous work in these laboratories[7]) the methyl group resonates at δ 3.01 ppm and the methine proton at δ 4.46 ppm: see Figure 5.

The ^{1}H NMR spectrum of anthraquinones **3** and **5** are shown in Figures 1 and 3 respectively.

2) Synthesis of an authentic sample of anthraquinone 4.

(a) Reaction of isopropylbenzene with phthalic anhydride.

Phthalic anhydride (14.8 g, 0.10 mol), aluminium trichloride (26.7 g, 0.20 mol) and isopropylbenzene (14.4 g, 0.12 mol) were reacted together following the general procedure. This gave acid **2** (24.4g, 91%), m.p. 130-133 °C (lit.,[3b] 129-130 °C); FT-IR ν_{max} (ATR): 2960, 1692, 1665 and 1603 cm^{-1}; ^{1}H NMR (500 MHz, $CDCl_3$) δ_{ppm} : 8.08 (dd, $^{3}J_{HH}$ = 7.9 Hz, $^{4}J_{HH}$ = 0.8 Hz, 1H), 7.66 (d, $^{3}J_{HH}$ = 8.4 Hz, 2H), 7.65 (td, $^{3}J_{HH}$ = 6.5 Hz, $^{4}J_{HH}$ = 1.2 Hz, 1H), 7.56 (td, $^{3}J_{HH}$ = 7.7 Hz, $^{4}J_{HH}$ = 1.1 Hz, 1H), 7.36 (dd, $^{3}J_{HH}$ = 7.5 Hz, $^{4}J_{HH}$ = 0.8 Hz, 1H), 7.28 (s, 1H), 2.95 (sept, $^{3}J_{HH}$ = 6.9 Hz, 1H), 1.26 ppm (d, $^{3}J_{HH}$ = 6.9 Hz, 6H).

(b) Cyclization of acid 2 to give 2-isopropylanthraquinone (4)

Acid **2** (10.0 g, 0.037 mol) was cyclized using the general procedure. This gave 2-iso-propylanthraquinone (**4**) (3.43 g, 37%), m.p. 44-46 °C (lit.,[3b] 45 °C); FT-IR ν_{max} (ATR): 2959, 1670, 1592 cm^{-1}; ^{1}H NMR (500 MHz, $CDCl_3$) δ_{ppm} : 8.35−8.27 (m, 2H), 8.24 (d, $^{3}J_{HH}$ = 8.0 Hz, 1H), 8.17 (d, $^{4}J_{HH}$ 1.6 = Hz, 1H), 7.82−7.76 (m, 2H), 7.66 (dd, $^{3}J_{HH}$ = 8.0 Hz, $^{4}J_{HH}$ = 1.7 Hz, 2H), 3.10 (sept, $^{3}J_{HH}$ = 6.9 Hz, 1H), 1.34 ppm (d, $^{3}J_{HH}$ = 7.0 Hz, 6H): see Figure 2.

Reference

6b. A. T. Peters and F. M. Rowe, *J. Chem. Soc.* **1945**, 181–182.

Figure 1: ^{1}H NMR spectrum of 1,3-diisopropylanthraquinone (**3**) in $CDCl_3$

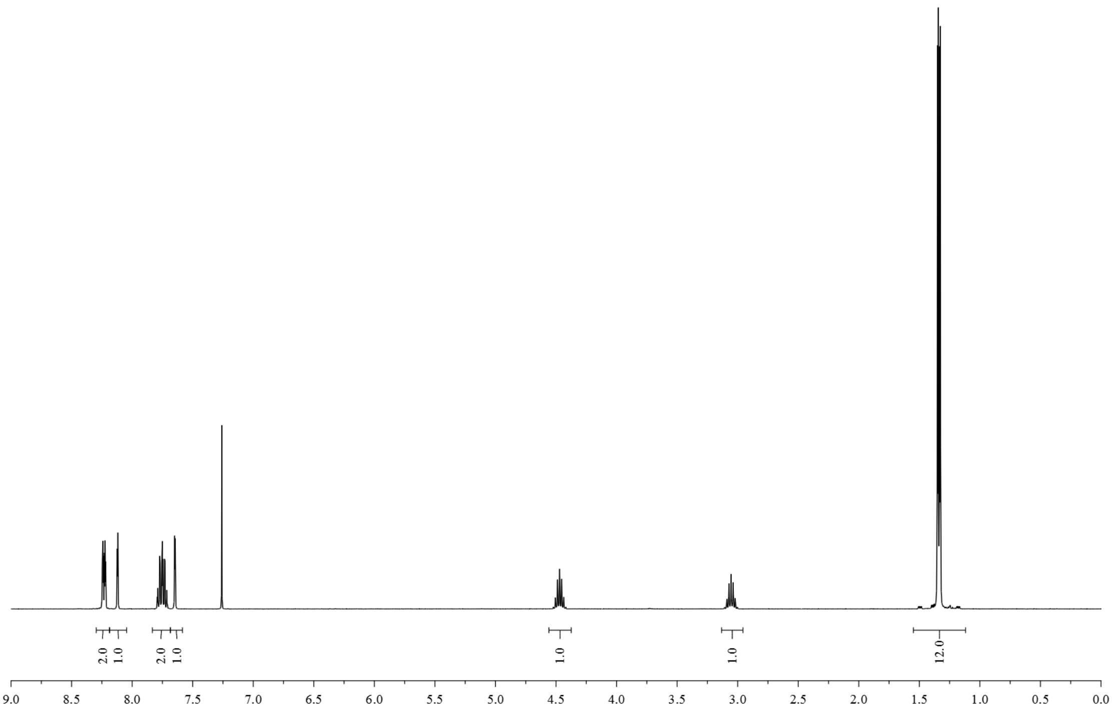

Figure 2: ^{1}H NMR spectrum of 2-isopropylanthraquinone (**4**) in $CDCl_3$

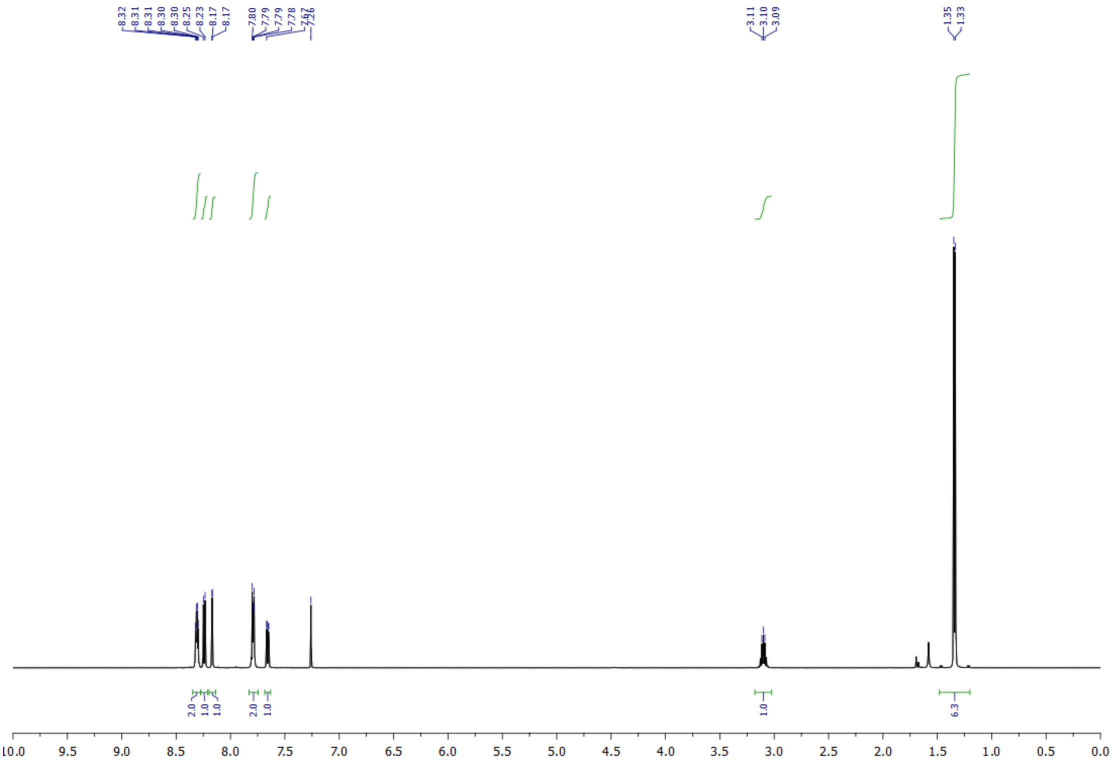

Figure 3: ^{1}H NMR spectrum of 1-methyl-3-isopropylanthraquinone (**5**) in $CDCl_3$

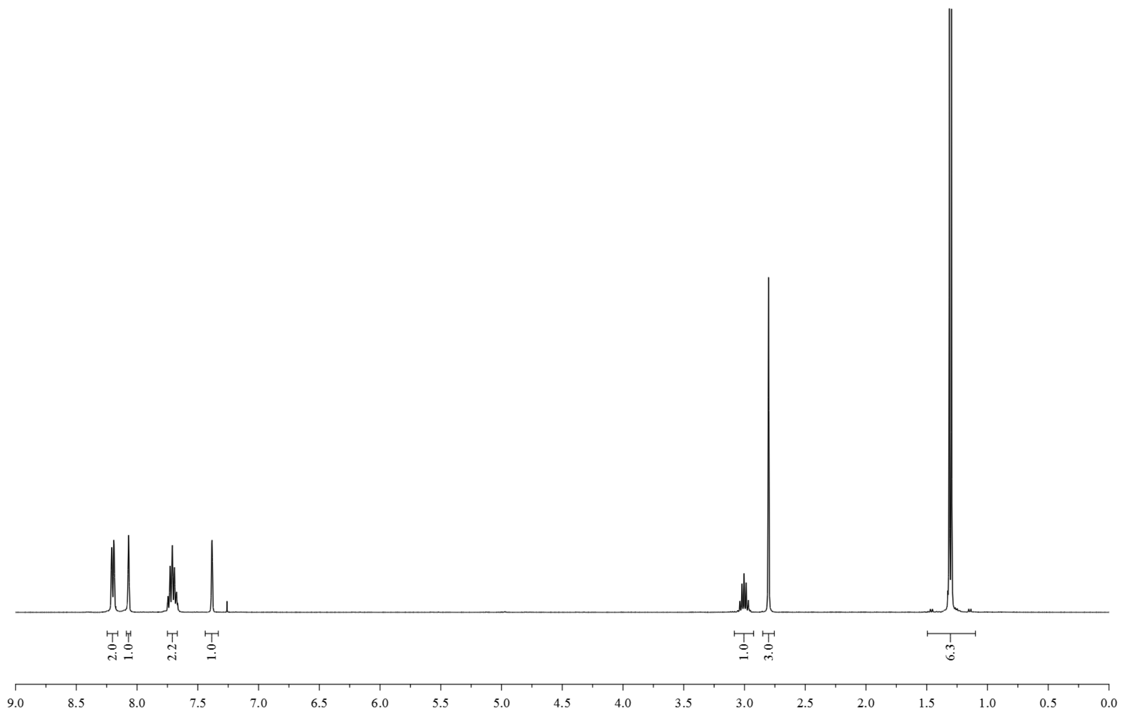

Figure 4: ^{1}H NMR spectrum of 2-methylanthraquinone (**6**) in $CDCl_3$

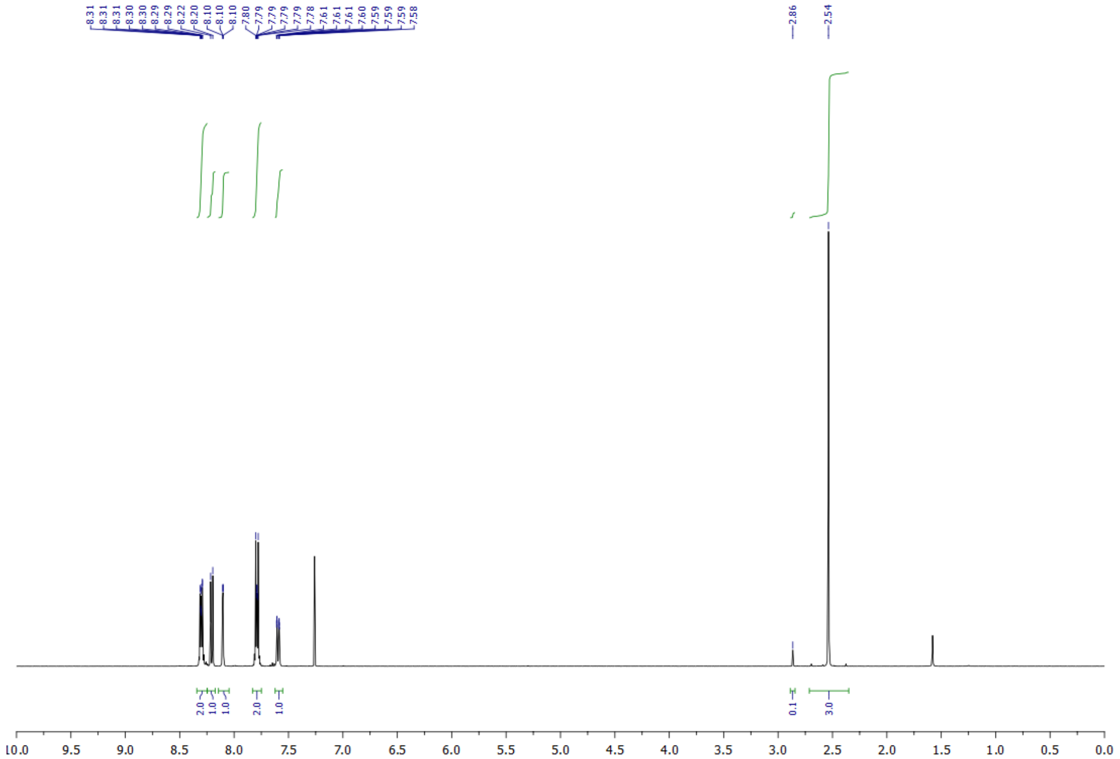

Figure 5: ^{1}H NMR spectrum of 1-isopropyl-4-methylanthraquinone (**7**) in $CDCl_3$.

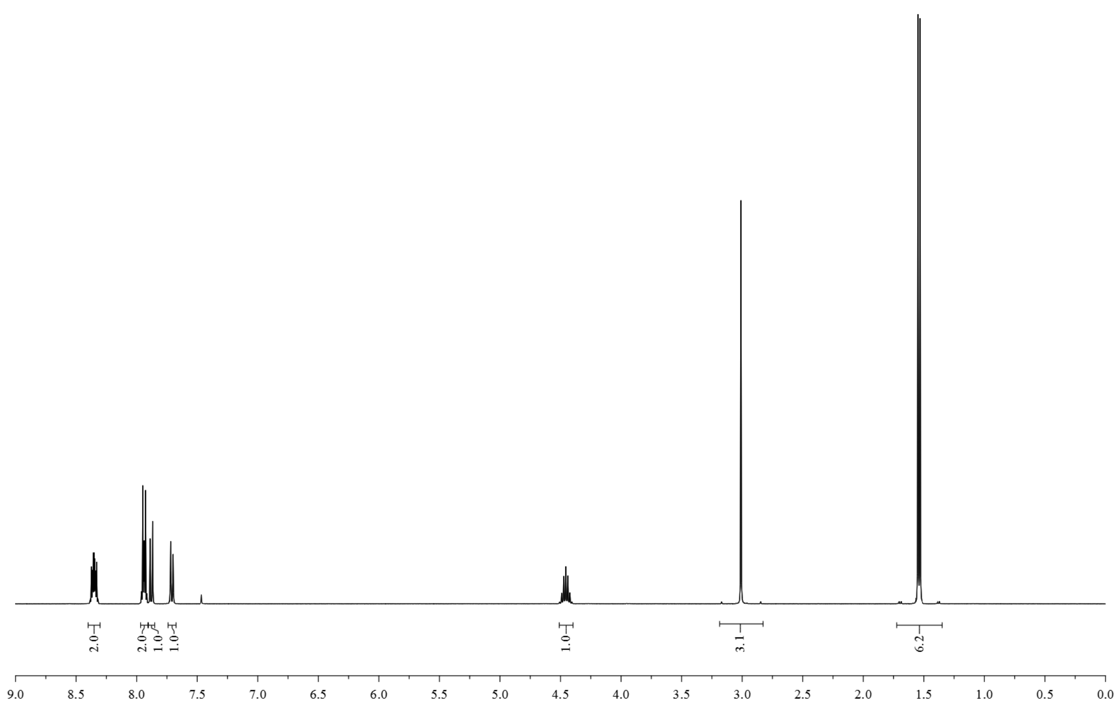

Supplement: As paper [file rsos170451supp1.pdf]
